# Phase I trial and pharmacological study of a 3-hour paclitaxel infusion in children with refractory solid tumours: a SFOP study

**DOI:** 10.1054/bjoc.2000.1637

**Published:** 2001-03

**Authors:** F Doz, J C Gentet, F Pein, D Frappaz, P Chastagner, S Moretti, G Vassal, J Arditti, O Van Tellingen, A Iliadis, J Catalin

**Affiliations:** Departement d'Oncologie Pédiatrique, Institut Curie, 26 rue d'Ulm, Paris, 75231 Cx 05; Service d'Oncologie Pédiatrique, CHU La Timone, Bd J. Moulin 13005 Marseille; Département d'Oncologie Pédiatrique, Institut Gustave Roussy Rue C. Desmoulins, 94800 Villejuif Cx; Service d'Oncologie pédiatrique, Centre Léon Bérard, 28 rue Laënnec 69000, Lyon; Service d'Hémato-cancérologie Pédiatrique, CHU Nancy, Hôpital d'Enfants de Brabois, 5 allée du Morvan, 54500 Vandoeuvreles Nancy; Centre anti-poison, 249 Bd Sainte Marguerite, Marseille, 13009; Department of Clinical Chemistry, The Netherlands Cancer Institute, Louwesweg 6, Amsterdam, 1066 CX; Faculté de Pharmacie, 27 av. J. Moulin, Marseille, 13385 Cx 051

**Keywords:** paclitaxel, short-term infusion, phase 1, children

## Abstract

The maximum tolerated dose of paclitaxel administered by 24-hour continuous infusion in children is known. Short infusion might offer equivalent antitumour efficacy and reduced haematological toxicity, without increasing the allergic risk. Our aims were to determine the maximum tolerated dose and the pharmacokinetics of paclitaxel in children when administered in 3-h infusion every 3 weeks. Patients older than 6 months, younger than 20 years with refractory malignant solid tumours were eligible when they satisfied standard haematological, renal, hepatic and cardiologic inclusion criteria with life expectancy exceeding 8 weeks. Paclitaxel was administered as a 3-hour infusion after premedication (dexamethasone, dexchlorpheniramine). Pharmacokinetic analysis and solvent assays (ethanol, cremophor) were performed during the first course. 20 courses were studied in 17 patients; 4 dosage levels were investigated (240 to 420 mg/m^2^). No dose-limiting haematological toxicity was observed. Severe acute neurological and allergic toxicity was encountered. One treatment-related death occurred just after the infusion at the highest dosage. Delayed peripheral neurotoxicity and moderate allergic reactions were also encountered. Pharmacokinetic analysis showed dose-dependent clearance of paclitaxel and elevated blood ethanol and Cremophor EL levels. Although no limiting haematological toxicity was reached, we do not recommend this paclitaxel schedule in children because of its acute neurological toxicity. © 2001 Cancer Research Campaign http://www.bjcancer.com


*British Journal of Cancer* (2001) **84**(5), 604–610
© 2001 Cancer Research Campaign
doi: 10.1054/ bjoc.2000.1637, available online at http://www.idealibrary.com on IDEAL

### Phase I trial and pharmacological study of a 3-hour paclitaxel infusion in children with refractory solid tumours: a SFOP study

**F Doz[1], JC Gentet[2], F Pein[3], D Frappaz[4], P Chastagner[5], S Moretti[1], G Vassal[3], J Arditti[6], O Van Tellingen[7], A Iliadis[8] and J Catalin[8]**

[1]Departement d'Oncologie Pédiatrique, Institut Curie, 26 rue d'Ulm, 75231 Paris Cx 05; [2]Service d'Oncologie Pédiatrique, CHU La Timone, Bd J. Moulin 13005 Marseille; [3]Département d'Oncologie Pédiatrique, Institut Gustave Roussy Rue C. Desmoulins, 94800 Villejuif Cx; [4]Service d'Oncologie pédiatrique, Centre Léon Bérard, 28 rue Laënnec 69000 Lyon; [5]Service d'Hémato-cancérologie Pédiatrique, CHU Nancy, Hôpital d'Enfants de Brabois, 5 allée du Morvan, 54500 Vandoeuvre les Nancy; [6]Centre anti-poison, 249 Bd Sainte Marguerite, 13009 Marseille; [7]Department of Clinical Chemistry, The Netherlands Cancer Institute, Louwesweg 6, 1066 CX Amsterdam; [8]Faculté de Pharmacie, 27 av. J. Moulin, 13385 Marseille Cx 051

**Summary** The maximum tolerated dose of paclitaxel administered by 24-hour continuous infusion in children is known. Short infusion might offer equivalent antitumour efficacy and reduced haematological toxicity, without increasing the allergic risk. Our aims were to determine the maximum tolerated dose and the pharmacokinetics of paclitaxel in children when administered in 3-h infusion every 3 weeks. Patients older than 6 months, younger than 20 years with refractory malignant solid tumours were eligible when they satisfied standard haematological, renal, hepatic and cardiologic inclusion criteria with life expectancy exceeding 8 weeks. Paclitaxel was administered as a 3-hour infusion after premedication (dexamethasone, dexchlorpheniramine). Pharmacokinetic analysis and solvent assays (ethanol, cremophor) were performed during the first course. 20 courses were studied in 17 patients; 4 dosage levels were investigated (240 to 420 mg/m$^2$). No dose-limiting haematological toxicity was observed. Severe acute neurological and allergic toxicity was encountered. One treatment-related death occurred just after the infusion at the highest dosage. Delayed peripheral neurotoxicity and moderate allergic reactions were also encountered. Pharmacokinetic analysis showed dose-dependent clearance of paclitaxel and elevated blood ethanol and Cremophor EL levels. Although no limiting haematological toxicity was reached, we do not recommend this paclitaxel schedule in children because of its acute neurological toxicity. © 2001 Cancer Research Campaign http://www.bjcancer.com

**Keywords**: paclitaxel; short-term infusion; phase 1; children

Paclitaxel is an anti-microtubular agent with a known efficacy in numerous adult solid tumours (Rowinsky and Donehower, 1995). A phase I trial of paclitaxel in children with refractory solid tumours showed that the recommended dose for phase II trials was 350 mg/m$^2$ day$^{-1}$ when administered as a 24-hour infusion. The main dose-limiting toxicity was neurological (peripheral neuropathy in one patient, tonic-clonic seizure in another). The other toxicities encountered were expected from adult experience: haematological toxicity, hypersensitivity (Hurwitz et al, 1993). However, some clinical data support the fact that a 3-hour short-term infusion of paclitaxel might offer equivalent anti-tumour efficacy and reduced haematological toxicity without increasing the risk of hypersensitivity (Eisenhauer et al, 1994). Phase I trials in adults of paclitaxel administered as a 3-hour infusion showed that the recommended dose for solid tumours was 210 mg/m$^2$ or 250 mg/m$^2$ with G-CSF support (Schiller et al, 1994; Younes et al, 1995).

Despite the usual remarkable chemosensitivity of malignant solid tumours in children, the development of new agents or new methods of drug administration with demonstrated efficacy and acceptable toxicity is still justified for refractory or relapsed tumours. The aim of this study was to determine the dose-limiting toxicity, maximum tolerated dose and pharmacokinetics of paclitaxel in children when administered as a 3-hour infusion. Since paclitaxel has low water solubility, it is formulated for clinical use in 50% cremophor EL and 50% ethanol, so that patients receiving paclitaxel therapy also receive a significant amount of these solvents. Because of the toxicity encountered, we also investigated the pharmacokinetics of these two solvents.

*Received 16 May 2000*
*Received 8 November 2000*
*Accepted 21 November 2000*

*Correspondence to:* F Doz

### PATIENTS AND METHODS

### Eligibility

Patients older than 6 months and younger than 20 years at the time of treatment with histologically documented malignant solid tumours, refractory to at least two lines of conventional therapeutic modalities with a life expectancy of more than 8 weeks were eligible for the study, provided they satisfied the following eligibility criteria: Lansky score ≥50 (Lansky et al, 1987), adequate haematological status (Granulocytes ≥1000 µl$^{-1}$, platelets ≥100 000 µl$^{-1}$ except in the case of bone marrow involvement), normal liver function (bilirubin ≤1.25 × N, AST and ALT ≤ 2 × N, fibrinogen ≤ 1.5 g l$^{-1}$ and prothrombin level ≥60%), adequate renal function (creatinine < 100 µmol l$^{-1}$, creatinine clearance ≥70 ml min$^{-1}$/1.73 m$^2$), normal electrocardiogram and echocardiography and no organ toxicity with WHO grade >2. In patients with epilepsy, the plasma level of the antiepileptic drug had to be within the therapeutic range. No other anticancer treatment was allowed during the study and the time interval between previous treatments and administration of paclitaxel had to be at least 3 weeks and 6 weeks in the case of previous use of nitrosoureas.

A patient could not be included in the case of severe infection, previous large field irradiation (such as craniospinal irradiation), previous use of high-dose chemotherapy with haematopoietic stem cell transplantation, known heart disease, previous neurological toxicity grade ≥2, disseminated hepatic metastases and known allergy to cremophor (e.g. previous allergy to teniposide). The protocol was approved by ethical committee and by participating institutions. Written informed consent was obtained from parents, guardians and children when appropriate.

### Treatment

Paclitaxel was supplied by Bristol Myers Squibb (Paris La Défense, France) as a concentrated sterile solution in 50% poly-oxy-ethylated castor oil (cremophor EL) and 50% dehydrated ethanol. Paclitaxel was diluted to a final concentration of 0.3 to 1.2 mg $ml^{-1}$ in 5% dextrose in water before administration over 3 hours with cardiac monitoring. Premedication was performed using dexamethasone (0.25 mg $kg^{-1}$, 12 and 6 hours before infusion) and dexchlorpheniramine (2.5 to 5 mg according to age) 30 minutes before infusion. No H2-blocking agent was used. The starting dose of paclitaxel was 240 mg/$m^2$ taking into account the recommended dose in adults for short-term infusion (Schiller et al, 1994) as well as the recommended dose in children for 24 hours infusion (Hurwitz et al, 1993). Dose escalation was planned with 20% increments: second step 290 mg/$m^2$, third step 350 mg/$m^2$, and fourth step 420 mg/$m^2$. A minimum of 3 patients was included at each step. There was no intra-patient dose escalation.

### Toxicity

Toxicity criteria were assessed after the first course, according to NCl-CTC and WHO definitions respectively. Toxicity was assessed after each course but dose-limiting toxicity was only assessed after the first course. The dose-limiting toxicity criteria were: persistence of grade IV neutropenia or thrombocytopenia for more than 7 days, any grade IV non-haematological toxicity, any grade III neurotoxicity, any grade III non-haematological toxicity except grade III nausea, grade III transitory hepatotoxicity, grade III fever and grade III mucositis. If at least two or more patients at a given dose level encountered one of these criteria, the dose-limiting toxicity (DLT) was reached and 3 additional patients were included to be treated at the dose level preceding the DLT.

Clinical examination, full blood count, liver, renal and electrolyte values, chest X-ray and antiepileptic drug assay, when applicable, were performed before each course. Haematological toxicity was assessed by at least 3 full blood counts per week. Except in the case of clinical evidence of disease progression after one course, tumour evaluation was planned to be performed every two courses. Patients could receive additional courses at 3-week intervals except in the case of disease progression or stable disease after 2 consecutive courses or dose-limiting toxicity. The eligibility criteria for further courses were the same as for the first course. The planned interval between two courses was 21 days with a maximum interval of 35 days.

### Pharmacokinetic studies

Blood samples were drawn before treatment and at 12 time points during and for up to 72 hours after infusion (90 and 180 minutes after the beginning of infusion; 10, 30, 60 minutes and 2, 4, 6, 12, 24, 48 and 72 hours after the end of infusion). Plasma was obtained by centrifugation and frozen at -20°C; 50μl to 1 ml of patient plasma were assayed for total paclitaxel determination by a validated high-performance liquid chromatography method (Martin et al, 1998). The limit of quantification was 25 ng $ml^{-1}$ (29 nM). In plasma spiked with 44, 440 and 750 ng $ml^{-1}$, the interassay variability was 11.18%, 2.97% and 3.02%, respectively. Total area under the curve (AUC) was determined by the linear trapezoidal rule extrapolated to infinity. The terminal rate constant (β) was determined by log-linear least-squares fit of the terminal elimination phase. Total-body clearance was calculated by dividing the dose by AUC. The $C_{max}$ sample was drawn at the end of infusion. Duration of threshold above 0.1 μM, which has been reported to be correlated with more severe neutropenia (Huizing et al, 1993; Gianni et al, 1995), has been determined in each patient. Plasma alcohol was assayed by a standard gas liquid chromatography method, using ethylacetate as internal standard. Cremophor EL levels were quantified by an HPLC method based on saponification of Cremophor EL, followed by extraction with chloroform and derivatization of the released fatty acid ricinoleic acid as described in detail previously (Sparreboom et al, 1996a) with minor modifications (Van Tellingen et al, 1999).

Correlation between observed toxicity and pharmacokinetic parameters of paclitaxel and solvents were studied.

### Tumour response

Response evaluation was planned after 2 courses and tumour response was defined as follows: complete response, complete regression of all apparent tumour masses; partial response, >50% decrease in the product of the greatest perpendicular diameters of all measurable lesions without appearance of new lesions; and minimal response, >25% but less than 50% objective decrease in measurable tumour without other evidence of disease progression. Stable disease was defined as a less than 25% objective decrease in a measurable tumour without other evidence of disease progression. Progressive disease was defined as a more than 25% increase in any measurable tumour. Evaluation of tumour was not planned after one course but a second course was not administered in the case of obvious clinical progression or severe toxicity of the first course.

### RESULTS

### Patient population

Between February 1995 and November 1995, 17 patients were included in the study and 4 dosage levels were investigated (Tables 1 and 2). 14 patients received only one course because of tumour progression or toxicity and 3 patients received 2 courses. No patients received more than 2 courses. The patient population consisted of 6 females and 11 males with a median age of 9 years (range: 19 months–19 years). Diagnoses are described in Table 1.

11 patients were treated for primary refractory disease and 6 patients for relapse. One to 4 chemotherapy lines were used before treatment with paclitaxel (median = 3) (Table 1). 8 patients had also received previous localized radiotherapy. At the time of

© 2001 Cancer Research Campaign 

**Table 1** Patients characteristics

| Characteristics | No of patients |
|---|---|
| Age, years | |
| Median | 9 |
| Range | 1.6–19 |
| Sex | |
| Male | 11 |
| Female | 6 |
| Tumour histology | |
| Rhabdomyosarcoma | 7 |
| Ewing's sarcoma | 2 |
| Neuroblastoma | 2 |
| Osteosarcoma | 1 |
| Hepatoblastoma | 1 |
| Nephroblastoma | 1 |
| Malignant glioma | 1 |
| Adrenocortical carcinoma | 1 |
| Kruckenberg tumour | 1 |
| No of prior therapy regimens | |
| 0–2 | 5 |
| 3–4 | 12 |
| Radiotherapy | 6 |

treatment, Lansky score was 100 for 12 patients, between 60 and 80 for 5 patients with correct general status but reduced mobility due to the tumour and 50 for one patient because of paraplegia. Only one patient received an anti-epileptic drug (carbamazepine) with a blood concentration within therapeutic range before treatment.

### Acute toxicity (Table 2)

We observed non-haematological dose-limiting toxicity in 2 patients after the first course at 420 mg/m$^2$ and another grade IV non-haematological toxicity in one patient after the second course at 350 mg/m$^2$. The first dose-limiting toxicity was observed in a patient who was included in the study for a refractory metastatic adrenocortical carcinoma treated with mitotane and hormonal substitution; her Lansky score was 100. She presented with nausea and headache 2 hours after starting the infusion followed by vomiting and diarrhoea during the third hour of infusion. No rash or oedema was observed. 30 minutes after the end of the infusion, she developed haemodynamic failure with brief cardiac arrest. She was rapidly managed with fluid modified gelatine, adrenaline and hydrocortisone hemisuccinate. Routine laboratory tests were performed while starting resuscitation and showed profound metabolic acidosis and no hypoglycaemia. Despite active management in the intensive care unit, she died as a result of multiorgan failure syndrome 12 hours after the start of paclitaxel infusion.

The other patient with dose-limiting toxicity had been included for a refractory malignant glioma and had a Lansky score of 100 before treatment. He developed transitory coma few minutes after the end of the infusion at 420 mg/m$^2$; there was no abnormal movement and the patient progressively recovered a normal level of consciousness within 4 hours. During the study, another patient experienced a similar neurological toxicity, but only after the second course. This patient, included at the 350 mg/m$^2$ level, was 19 months old and weighed 9.8 kg. Because of his young age and low weight, and because of the previous toxicity observed at the 420 mg/m$^2$ step, he received the first course at 2/3 of the theoretical dose (equivalent to 250 mg/m$^2$) and had not experienced any limiting toxicity. This patient then received a second course at the full dose level and we observed transitory coma following this second paclitaxel infusion. In both patients with transient coma, routine laboratory tests were performed. No hypoglycaemia was observed but transitory metabolic acidosis was found in both cases (pH 7.26 and 7.23; bicarbonate 16 and 8 mmol l$^{-1}$ respectively); transient elevation of transaminases (5 × N) for 4 days in one of these 2 patients was also noted. Electro-encephalogram was performed in these 2 cases and showed no specific abnormality.

One grade I central nervous system toxicity was also observed with transitory alteration of consciousness in one patient receiving concomitant morphine dose escalation. In two patients, transitory grade I and II alteration of consciousness (anxiety or hypersomnia)

**Table 2** Neurological and allergic toxicity after paclitaxel infusion

| | Patient number | Dosage (mg m$^{-2}$) | Neurological toxicity: Acute | Grade | Delayed | Grade | Allergic toxicity: Acute | Grade | Delayed | Grade |
|---|---|---|---|---|---|---|---|---|---|---|
| | 201 | 290 | | | | | | | | |
| | 202 | 290 | | | paraesthesia | I | | | rash/pruritis | I |
| II | 203 | 290 | | | dysaesthesia | III | | | rash/pruritis | I |
| | 204 | 290 | mild agitation | I | excitation | I | | | rash/pruritis | I |
| | 205 | 290 | | | | | | | | |
| | 206 | 290 | | | | | | | | |
| | 301 | 350 | somnolence[b] | I | | | | | | |
| IIIa | 302 | 350 | | | headache, ileus | | | | | |
| | 303 | 350 | | | and paraesthesia | II | | | | |
| | 311 | 250[a] | | | | | | | | |
| *IIIb* | *311* | *350* | *coma* | *IV* | | | | | | |
| | 312 | 350 | somnolence/agitation | II | | | mild pruritis | I | rash/pruritis | II |
| | 401 | 420 | coma | IV | dysaesthesia | III | | | | |
| IV | 402 | 420 | | | | | | | | |
| | 403 | 420 | coma/agitation | IV | | | anaphylactic collapse/death | IV | | |

Toxicity was assessed according to the NCI-CTC definitions. [a]Patient no. 311, step IIIB was 19 months old and weighed 9.8 kg: he received the first course at 2/3 of the theoretical dose (equivalent to 250 mg/m$^2$) and no limiting toxicity was observed. This patient then received a second course at the full dose level (line in *italics*) and transitory coma was observed following this second paclitaxel infusion. [b]patient no. 301: had morphine dose escalation just before paclitaxel infusion.

 *© 2001 Cancer Research Campaign*

were noted during the infusion. No neurological toxicity was observed in the patient who received carbamazepine anticonvulsive therapy. Grade I or II nausea was observed in three courses at 290 and 350 mg/m$^2$.

### Delayed toxicity (Table 2)

The observed haematological toxicity was not dose limiting. Grade IV neutropenia was observed twice at the 240 and 350 mg/m$^2$ dosage levels, with a duration less than 7 days, and grade III neutropenia was observed 5 times (at 290 and 350 mg/m$^2$). No grade IV leukopenia occurred; grade III leukopenia occurred 5 times at 240, 290 and 350 mg/m$^2$. No thrombocytopenia was encountered. Red blood cell transfusions were necessary in 4 courses for 3 patients receiving 290, 350 and 420 mg/m$^2$; 2 of these patients had an intra-tumour bleeding. Only one patient experienced documented infection with *Enterobacter* septicaemia during grade IV neutropenia (stage 4 neuroblastoma). 2 patients experienced transitory fever of unknown origin without neutropenia at 350 mg/m$^2$.

Delayed hypersensitivity reactions with skin rash and pruritis were observed 4 times at 290 and 350 mg/m$^2$. These symptoms persisted for 7 to 19 days. One case of pruritis was observed 24 hours after the end of the infusion, which then persisted for 19 days associated with skin rash (grade II). In one patient, at the 350 mg/m$^2$ dose, these symptoms occurred very intensively between days 10 and 15 after the infusion and required hospitalization and treatment with antihistaminic drugs, corticosteroids, benzodiazepine and morphine. Another patient presented paraesthesia and grade III pruritis, but without skin rash, between day 5 and day 15 after a 420 mg/m$^2$ infusion, requiring hospitalization and was successfully treated by clonazepam, sulpiride and corticosteroids: we interpreted these symptoms as a peripheral nervous system toxicity rather than an allergic reaction.

No mucositis was observed. One patient experienced grade I delayed vomiting at 290 mg/m$^2$. One patient experienced transitory grade II diarrhoea, two days after the infusion at 350 mg/m$^2$. One patient also experienced transitory ileus at 350 mg/m$^2$, but with concomitant increasing morphine dosage. No late renal or electrolyte abnormality was observed. Elevated transaminases 3 weeks after the infusion were recorded 3 times at the 290, 350 and 420 mg/m$^2$ dosage levels to values 2 to 13 times normal values. Alopecia was observed 3 times in 3 evaluable patients.

### Pharmacokinetics and correlation with toxicity

Pharmacokinetic data were available for all 17 patients. The pharmacokinetic analysis of the 19-month-old patient who was included at the 350 mg/m$^2$ dose level but treated during the first course at 2/3 of the theoretical dose (equivalent to 250 mg/m$^2$ for a weight of 9.8 kg) was performed only during this first course: no pharmacokinetic analysis was performed during the second course at the full dose level associated with grade IV neurological toxicity. Selected pharmacokinetic parameters of paclitaxel are listed in Table 3 together with the $C_{max}$ values for ethanol and cremophor. Figure 1 illustrates the typical pharmacokinetic profiles of paclitaxel at 290 mg/m$^2$. For each dose level, mean duration of paclitaxel plasma concentration threshold above 0.1 µM was respectively 21, 24, 30 and 34 hours. In our study, we observed high plasma alcohol levels after paclitaxel infusion: $C_{max}$ values of ethanol ranged between 0.24 and 2.04 g l$^{-1}$ (Table 3). Furthermore, the $C_{max}$ concentrations of cremophor in plasma after paclitaxel infusions ranged between 6.12 and 22.37 g l$^{-1}$ (Table 3). We retrospectively calculated that the amount of ethanol received by the patients over 3 hours ranged between 0.39 and 1.05 g kg$^{-1}$ and the amount of cremophor ranged between 0.52 and 1.4 g kg$^{-1}$. Severe neurological toxicity was observed in both patients with the highest concentrations of ethanol and cremophor. 3 patients with coma and somnolence in whom ethanol concentration was measured have 3 of the 4 highest ethanol concentrations, above 1.45 g l$^{-1}$. No clear correlation of the toxicity with age was observed.

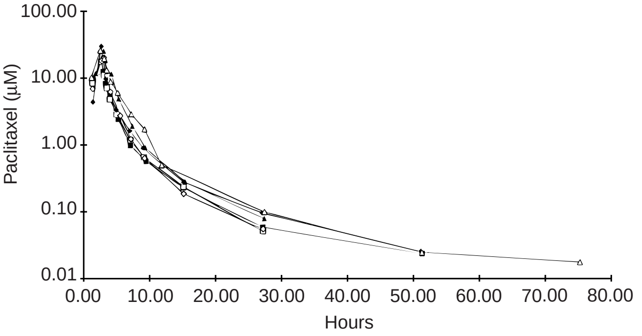

**Figure 1** Plasma concentrations of paclitaxel in children treated with 290 mg/m$^2$ of paclitaxel as a 3-hour infusion

**Table 3** Pharmacokinetics of paclitaxel in children after 3-hour infusion. Determination of $C_{max}$ of the solvents, ethanol and cremophor. Means and standard deviation.

| | Paclitaxel data | | | | | Alcool | Cremophor |
|---|---|---|---|---|---|---|---|
| Dose level mg/m$^2$ | Cmax µM | Clexp L/h/m$^2$ | AUCexp µMxh | T1/2elim H | Vβ L | $C_{max}$ (g l$^{-1}$) | $C_{max}$ (g l$^{-1b}$) |
| 240 | 8.3[a] | 8.48 | 33.8 | 7.23 | 140.8 | 0.40[a] | 8.37[a] |
| | (2.1) | (1.24) | (4.8) | (1.34) | (31.1) | (0.15) | (2.05) |
| 290 | 22.4 | 4.99 | 70.8 | 9.69 | 71.7 | 0.93 | 13.15 |
| | (6.5) | (0.99) | (15.7) | (4.93) | (38.6) | (0.24) | (2.29) |
| 350 | 40.2 | 3.39 | 156.7 | 5.23 | 19.2 | 1.17 | 16.9 |
| | (17.8) | (1.61) | (75.7) | (1.36) | (9.1) | (0.39) | (3.4) |
| 420 | 38.9[a] | 2.74 | 179.6 | 10.24 | 39.4 | 1.6[a] | 18.9[a] |
| | (4.9) | (0.01) | (0.1) | (0.8) | (8.3) | (0.38) | (3.6) |

[a]$C_{max}$ under-estimated in 2 cases because infusion duration greater than 3 hours. [b]1 g l$^{-1}$ = 0.95 µl ml$^{-1}$

© 2001 Cancer Research Campaign 

### Tumour responses

Tumour response was evaluable in all patients, except for one case because of early death. One patient treated for embryonal rhabdomyosarcoma experienced a more than 50% decrease of an abdominal lymph node after one course at 350 mg/m$^2$, but treatment had to be stopped because of a delayed allergic reaction. Stable disease was observed after 2 courses at 350 mg/m$^2$ in one patient with alveolar rhabdomyosarcoma but the treatment had to be stopped because of a severe toxicity at the second course. For 2 other patients, we did not observe disease progression after one course but treatment had to be stopped because of severe toxicity. Disease progression was observed in the 12 other evaluable patients (10 after 1 course, 2 after 2 courses).

### DISCUSSION

The haematological toxicity was not dose limiting in this study, despite high paclitaxel $C_{max}$ and prolonged time of paclitaxel plasma levels above 0.1 μM which is usually correlated to more severe neutropenia (Huizing et al, 1993; Gianni et al, 1995). This low haematological toxicity, associated with apparent high drug exposure, might be due to a better haematological tolerance of paclitaxel in children than in adults. However, this might also be due to interference between the paclitaxel and cremophor EL pharmacokinetics: it has been recently reported that the higher plasma levels of paclitaxel do not reflect higher levels in tissues, since cremophor EL increases the affinity of paclitaxel to plasma components (Van Tellingen et al, 1999).

On the other hand, acute non-haematological toxicity was dose limiting and related to high $C_{max}$ of paclitaxel, ethanol and cremophor EL. Severe acute toxicity were observed in 2 patients after the first course given at a dose of 420 mg/m$^2$ and also in an infant who received the full dose of 350 mg/m$^2$ for the second course. The origin of the toxic death observed at the fourth dosage level is difficult to determine: the initial symptoms and clinical history are concordant with severe allergic reaction and/or acute neurological toxicity, occurring in a debilitated patient dependent on hydrocortisone replacement therapy. Similar toxicity was not observed when the same dosages were given to children over 24 hours. However, the central nervous system toxicity of paclitaxel has already been reported: seizures in children (Hurwitz et al, 1993), alteration of consciousness (Webster et al, 1996; Chang et al, 1998; Glück et al, 1998) or neurovegetative disorders (Vassilomanolakis et al, 1998) in adults. The mechanism of acute neurological toxicity is not completely understood. The well known peripheral nervous system toxicity (Hurwitz et al, 1993; Cavaletti et al, 1995; Seidman et al, 1995; Glantz et al, 1996), also observed in our study, might reflect a direct axonal toxicity, that may also affect the central nervous system. However, brain penetration of paclitaxel is known to be limited (Eiseman et al, 1994; Glantz et al, 1995). Nevertheless, grade IV neurological toxicity was encountered for the highest plasma concentrations and AUC of paclitaxel and the lowest clearances. The role of ethanol, used as paclitaxel solvent, must also be stressed in the acute neurological toxicity. Ethanol causes cardiovascular and central nervous system toxicity, direct vasodilatation (Litovitz, 1986) and hypoglycaemia as well as metabolic acidosis (Ellenhorn, 1997). In our study, transitory metabolic acidosis but no hypoglycaemia was observed in both patients who experienced transitory coma at the end of the infusion; in one patient, a transitory increase of transaminases was observed (5 × N) but with no other hepatic abnormality. Ethanol is used as a solvent for other cytotoxic drugs such as etoposide or BCNU. The quantity of ethanol delivered with etoposide in a CARBOPEC regimen (Namouni et al, 1997) is 0.14 g kg$^{-1}$ day$^{-1}$ in 1 hour from D1 to D5. The quantity of ethanol delivered with BCNU in a BEAM regimen (Gaspard et al, 1988) is 0.3 g kg$^{-1}$ in 1 hour. At 420 mg/m$^2$ dose level, the quantity of ethanol delivered with paclitaxel in this study was 1.05 g kg$^{-1}$ in 3 hours. Lethal doses of ethanol in children are known to be around 3 g kg$^{-1}$ but this is mainly reported after oral intoxication. Blood concentrations above 3 g l$^{-1}$ are known to be potentially lethal. Ethanol assays in plasma samples collected for pharmacokinetic analysis showed elevated blood ethanol levels compatible with acute reversible neurological toxicity, but not necessarily explaining the toxic death (Moss, 1970; Sellers and Kalant, 1976; Landers, 1983; Adinoff et al, 1988). Somnolence and coma may be correlated partly with ethanol exposure (high $C_{max}$ and duration of plasma alcohol concentrations above 0.5 g l$^{-1}$). Ethanol assays have also been reported in adults treated with paclitaxel. In one report (Webster et al, 1996), ethanol $C_{max}$ after 175 mg/m$^2$ of paclitaxel did not exceed 0.09 g l$^{-1}$ in 10 out of 12 patients, but reached values of 0.17 and 0.33 g l$^{-1}$ in 2 other patients. Wilson (Wilson et al, 1997) reported a case of ethanol intoxication associated with a 3-hour dose of 350 mg/m$^2$ paclitaxel infusion, leading to an ethanol concentration of 0.98 g l$^{-1}$. Finally, the role of cremophor in acute neurological toxicity must also be discussed, as cremophor has been shown in vitro to induce demyelinization and axonal swelling and degeneration (Windenbank et al, 1994) at levels that are largely achieved in vivo using 3-hour paclitaxel infusions. In any case, in view of the acute neurological toxicity and elevated plasma alcohol concentrations which had not been anticipated and were not associated with any dose-limiting haematological toxicity, we considered that this paclitaxel schedule was definitely not recommended in children. Indeed, because of the suspected role of solvents in this non-haematological severe toxicity, the study was closed without including the 2 more patients at the third dose level.

The other main toxicity encountered in this study was allergic. Allergy to paclitaxel is usually thought to be due to cremophor and is generally successfully prevented by anti-allergic premedication. Cremophor has been implicated as a probable cause of anaphylactoid reactions following administration of cremophor-containing drugs, such as cyclosporin or teniposide (Chapuis et al, 1985; Magalini et al, 1986; MacLeod et al, 1991; Theis et al, 1995; Nolte et al, 1998). Such a severe allergic reaction might also be involved in the origin of the toxic death that occurred in our study. However, given the allergic toxicity described with the analogue docetaxel (Seibel et al, 1999), which is delivered without cremophor EL, paclitaxel itself might also be involved in these allergic reactions. The use of an $H_2$-blocking agent has been recommended before paclitaxel infusion in adults (Schiller et al, 1994), but remains controversial and was not used in the previous phase I trial in children (Hurwitz et al, 1993).

The pharmacokinetics of paclitaxel in 3 hour infusion showed specific characteristics. Clearance of paclitaxel appeared to decrease as the dosage increased from 240 to 420 mg/m$^2$ and the maximum concentration and area under the curve increased more than in proportion to the dose. As in adults (Eisenhauer et al, 1994; Sonnichsen et al, 1994a; Gianni et al, 1995; Kearns et al 1995), these results suggest that paclitaxel pharmacokinetics is non-linear when the drug is administered as a 3-hour infusion in children.

 © 2001 Cancer Research Campaign

Mean paclitaxel clearances in children (8.48 to 2.74 l $h^{-1}/m^2$) were within the range reported for adults after short-term administration (Sonnichsen et al, 1994a; Gianni et al, 1995; Kearns et al, 1995) but clearances tended to be lower after the 3-hour infusion (our study) compared to the 24-hour infusion value in children (Sonnichsen et al, 1994b). However, although it has previously been proposed that paclitaxel's nonlinear pharmacokinetic behaviour is due to saturable distribution and elimination processes, recent reports strongly suggest that this non-linear behaviour results from entrapment of paclitaxel in cremophor EL micelles in plasma (Sparreboom et al, 1996b, 1999; Van Tellingen et al, 1999). This effect makes it difficult to use the clearance as parameter for drug exposure, as the actual exposure to 'free' paclitaxel depends on the plasma level of cremophor EL. The pharmacokinetics of cremophor EL itself was only studied in adults (Rischin et al, 1996; Sparreboom et al, 1999; Van Tellingen et al, 1999). These studies have shown that the $C_{max}$ of cremophor EL increases linearly with the dose administered. At the highest dose level tested in adults (Sparreboom et al, 1999) of 225 mg/$m^2$, the $C_{max}$ of cremophor EL was $6.58 \pm 0.52$ µl $ml^{-1}$ (i.e. 6.94  0.55 mg $ml^{-1}$), which is comparable to the levels found in our study in children receiving the lowest dose of 240 mg/$m^2$. Further increments in the dose given to children resulted in a linear increase in the cremophor EL $C_{max}$ levels. Consequently, the $C_{max}$ level of cremophor EL at the highest dose (420 mg/$m^2$) is more than 2-fold higher than in adults receiving conventional doses of paclitaxel, which might explain the apparent non-linearity of paclitaxel's clearance and the low haematological toxicity.

Regarding anti-tumour activity, the only tumour response in this study was obtained in one patient treated for recurrent rhabdomyosarcoma. It might be interesting to investigate other paclitaxel schedules in this diagnosis (Hurwitz et al, 1993).

In conclusion, dose-limiting toxicity of this modality of paclitaxel administration in children, is neurological and, possibly, allergic. The combination of paclitaxel, ethanol and cremophor EL is responsible for neurotoxicity; since all components may play a role, it does not seem appropriate to relate toxicity to specifically one component. Paclitaxel administration as a 3-hour infusion every 3 weeks is definitely not recommended in children. Short-term infusions may be used for administration at lower doses, such as weekly (Glantz et al, 1996) or fractionated daily doses (Don Francesco et al, 1996). In addition, plasma alcohol levels should be monitored in children receiving paclitaxel at any dose level.

### ACKNOWLEDGEMENTS

The authors thank Dr V Mosseri (Biostatistics Department, Institut Curie, Paris) for her help in the design of the study; Bristol Myers Squibb (Paris la Défense), for helping in the design of the study, providing the drug, supporting data collection and pharmacokinetic analysis; Pr Philippe Hubert regarding information about ethanol toxicity; D Bours for her help in data collection and I Dubois-Noël for typing the manuscript.

### REFERENCES

Adinoff B, Bone GH and Linnoila M (1988) Acute ethanol poisoning and the ethanol withdrawal syndrome. *Med Toxicol* **3**: 172–196

Cavaletti G, Bogliun G, Marzorati L, Zincone A, Marzola M, Colombo N and Tredici G (1995). Peripheral neurotoxicity of Taxol in patients previously treated with Cisplatin. *Cancer* **75**: 1141–1150

Chang SM, Kuhn JG, Rizzo J, Robins HI, Schold SC Jr, Spence AM, Berger MS, Mehta MP, Bozik ME, Pollack I, Gilbert M, Fulton D, Rankin C, Malec M and Prados MD (1998) Phase I study of paclitaxel in patients with recurrent malignant glioma: a North American brain tumor consortium report. *J Clin Oncol* **16**: 21888–22194

Chapuis B, Helg C, Jeannet M, Zulian G, Huber P and Gumovski P (1985) Anaphylactic reaction to intravenous cyclosporine. *N Englj Med* **312**: 1259

Donfrancesco A, Deb G, De Sio L, De Laurentis C, Fidani P, Cozza R, Jenkner A, Castellano A and Habetswallner D (1996) Phase I–II trial of Taxol according to Q4D regimen in pediatric patients with recurrent solid tumors. *Med Pediatr Oncol* **27**: 211 (abstr O2)

Eiseman JL, Eddington ND, Leslie J, MacAuley C, Sentz DL, Zuhowski M, Kujawa JM, Young D and Egorin MJ (1994) Plasma pharmacokinetics and tissue distribution of paclitaxel in $CD_2F_1$ mice. *Cancer Chemother Pharmacol* **34**: 465–471

Eisenhauer EA, ten Bokkel Huinink WW, Swenerton KD, Gianni L, Myles J, van deBurg MEL, Kerr I, Vermorken JB, Buser K, Colombo N, Bacon M, Santabarbara P, Onetto N, Winograd B and Canetta R (1994) European-Canadian randomized trial of paclitaxel in relapsed ovarian cancer: high-dose versus low-dose and long versus short infusion. *J Clin Oncol* **12**: 2654–2666

Ellenhorn M (1997) Alcohols and glycols. In: *Medical toxicology*, 2nd edn, Ellenhorn (ed) pp. 1127–1147. William and Wilkins: Baltimore

Gaspard MH, Maraninchi D, Stoppa AM, Gastaut JA, Michel G, Tubiana N, Blaise D, Novakovitch G, Rossi JF, Weiller PJ, Sainty D, Horchowski N and Carcassone Y (1988) Intensive chemotherapy with high doses of BCNU, Etoposide, Cytosine Arabinoside and Melphalan (BEAM) followed by autologous bone marrow transplantation: toxicity and antitumor activity in 26 patients with poor-risk malignancies. *Cancer Chemother Pharmacol* **22**: 256–262

Gianni L, Kearns CM, Giani A, Capri G, Vigano L, Lacatelli A, Bonadonna G and Egorin MJ (1995) Nonlinear pharmacokinetics and metabolism of paclitaxel and its pharmacokinetic/pharmacodynamic relationships in humans. *J Clin Oncol* **13**: 180–190

Glantz MJ, Choy H, Kearns CM, Mills PC, Wahlberg LU, Zuhowski EG, Calabresi P and Egorin MJ (1995) Paclitaxel disposition in plasma and central nervous system of humans and rats with brain tumors. *J Natl Cancer Inst* **87**: 1077–1081

Glantz MJ, Choy H, Kearns CM, Cole BF, Mills P, Zuhowski EG, Saris S, Rhodes CH, Stopa E and Egorin MJ (1996) Phase I study of weekly outpatient Paclitaxel and concurrent cranial irradiation in adults with astrocytomas. *J Clin Oncol* **14**: 600–609

Gluck S, Germond C, Lopez P, Cano P, Dorreen M, Koski T, Arnold A, Dulude H and Gallant G (1998) A phase I trial of high-dose paclitaxel, cyclophosphamide and mitoxantrone with autologous blood stem cell support for the treatment of metastatic breast cancer. *Eur J Cancer* **34**: 1008–1014

Huizing MT, Keung AC, Rosing H, van der Kuij V, ten Bokkel Huinink WW, Mandjes IM, Dubbelman AC, Pinedo HM and Beijnen JH (1993) Pharmacokinetics of paclitaxel and metabolites in a randomized comparative study in platinum-pretreated ovarian cancer patients. *J Clin Oncol* **11**: 2127–2135

Hurwitz CA, Relling MV, Weitman SD, Ravindranath Y, Vietti TJ, Strother DR, Ragab AH and Pratt CB (1993) Phase I trial of Paclitaxel in children with refractory solid tumors: a pediatric oncology group study. *J Clin Oncol* **11**: 2324–2329

Kearns CM, Gianni L and Egorin MJ (1995). Paclitaxel pharmacokinetics and pharmacodynamics. *Sem Oncol* **22**: 16–23

Landers DF (1983) Alcoholic coma and some associated conditions. *Am Fam Physician* **28**: 219–222

Lansky SB, List MA, Lansky LL, Ritter-Sterr C and Miller DR (1987) The measurement of performance in childhood cancer patients. *Cancer* **60**: 1651–1656

Litovitz T (1986) The alcohols: ethanol, methanol, isopropanol, ethylene glycol. *Pediatr Clin North Am* **33**: 311

McLeod HL, Baker DK Jr, Pui CH and Rodman JH (1991) Somnolence, hypotension and metabolic acidosis following high-dose teniposide in children with leukaemia. *Cancer Chemother Pharmacol* **29**: 150–154

Magalini SC, Nanni G, Agnes S, Citterio F and Castagneto M (1986) Anaphylactic reaction to first exposure to cyclosporine. *Transplantation* **42**: 443–444

Martin N, Catalin J, Blachon MF and Durand A (1998) Assay of paclitaxel (Taxol) in plasma and urine by high-performance liquid chromatography. *J Chromatogr Biomed Applic* **709**: 281–288

Moss MH (1970) Alcohol-induced hypoglycemia and coma caused by alcohol sponging. *Pediatrics* **46**: 445–447

© 2001 Cancer Research Campaign 

Namouni F, Doz F, Tanguy ML, Quintana E, Michon J, Pacquement H, Bouffet E, Gentet JC, Plantaz D, Lutz P, Vannier JP, Validire P, Neuenschwander S, Desjardins L and Zucker JM (1997) High dose chemotherapy with Carboplatin, Etoposide and Cyclophosphamide followed by hematopoietic stem cell rescue in patients with high risk retinoblastoma: a SFOP and SFGM study. *Eur J Cancer* **33**: 2368–2375

Nolte H, Carstensen H and Hertz H (1988). VM-26 (teniposide)-induced hypersensitivity and degranulation of basophils in children. *Am J Pediatr Hematol Oncol* **10**: 308–312

Rischin D, Webster LK, Millward MJ, Linahan BM, Toner GC, Woollett AM, Morton CG and Bishop JF (1996) Cremophor pharmacokinetics in patients receiving 3-, 6- and 24-hour infusions of paclitaxel. *J Natl Cancer Inst* **18**: 1297–1301

Rowinsky EK and Donehower RC (1995) Paclitaxel (Taxol). *N Engl J Med* **332**: 1004–1014

Schiller JH, Storer B, Tutsch K, Arzoomanian R, Alberti D, Feierabend C and Spriggs D (1994) Phase I trial of 3-hour infusion of paclitaxel with or without granulocyte colony-stimulating factor in patients with advanced cancer. *J Clin Oncol* **12**: 241–248

Seibel NL, Blaney SM, O'Brien M, Krailo M, Hutchinson R, Mosher RB, Balis FM and Reaman GH (1999) Phase I trial of docetaxel with filgrastim support in pediatric patients with refractory solid tumors: a collaborative Pediatric Oncology Branch, National Cancer Institute and Children's Cancer Group trial. *Clin Cancer Res* **5**: 733–737

Seidman AD, Tiersten A, Hudis C, Gollub M, Barrett S, Yao TJ, Lepore J, Gilewski T, Currie V, Crown J, Kakes T, Baselga J, Sklarin N, Moynihan ME, Tong W, Egorin M, Kearns C, Spriggs D and Norton L (1995) Phase II trial of paclitaxel by 3-hour infusion as initial and salvage chemotherapy for metastatic breast cancer. *J Clin Oncol* **13**: 2575–2581

Sellers EM and Kalant H (1976) Alcohol intoxication and withdrawal. *N Engl J Med* **294**: 757–762

Sonnichsen DS and Rellin MV (1994) Clinical pharmacokinetics of paclitaxel. *Clin Pharmacokinet* **27**: 256–269

Sonnichsen DS, Hurwitz CA, Pratt CB, Shuster JJ and Relling MV (1994) Saturable pharmacokinetics and Paclitaxel pharmacodynamics in children with solid tumors. *J Clin Oncol* **12**: 532–538

Sparreboom A, Van Tellingen O, Huizing MT, Nooijen WJ and Beijnen JH (1996a) Determination of polyoxyethyleneglycerol triricinoleate 35 (Cremophor EL) in plasma by pre-column derivatization and reversed-phase high-performance liquid chormatography. *J Chromatogr B Biomed Appl* **681**: 355–361

Sparreboom A, van Tellingen O, Nooijen WJ and Beijnen JH (1996b) Nonlinear pharmacokinetcs of paclitaxel in mice results from the pharmaceutical vehicle cremophor EL. *Cancer Res* **56**: 2112–2115

Sparreboom A, van Zuylen L, Brouwer E, Loss WJ, de Bruijn P, Gelderblom H, Pillay M, Nooter K, Stoter G and Verweij J (1999) Cremophor EL-mediated alteration of paclitaxel distribution in human blood: clinica pharmacokinetic implications. *Cancer Res* **59**: 1454–1457

Theis JG, Liau-Chu M, Chan HS, Doyle J, Greenberg ML and Koren G. (1995) Anaphylactoid reaction in children receiving high-dose intravenous cyclosporine for reversal of tumor resistance: the causative role of improper dissolution of Cremophor EL. *J Clin Oncol* **13**: 2508–2516

van Tellingen O, Huizing MT, Panday VR, Schellens JH, Nooijen WJ and Beijnen JH (1999) Cremophor EL causes (pseudo-) non linear pharmacokinetics of paclitaxel in patients. *Br J Cancer* **81**: 330–335

Vassilomanolakis M, Tsoussis S and Efremedis A (1998) Long lasting, grade IV, orthosatic hypotension after a single cycle combination chemotherapy with paclitaxel and cisplatin. *Eur J Cancer* **34**: 1295

Webster LK, Crinis NA, Morton CG and Millward MJ (1996) Plasma alcohol concentrations in patients following paclitaxel infusion. *Cancer Chemother Pharmacol* **37**: 499–501

Wilson DB, Beck TM and Gundlach CA (1997) Paclitaxel formulation as a cause of ethanol intoxication. *Ann Pharmacother* **31**: 873–875

Windebank AJ, Blexrud MD and De Groen PC (1994) Potential neurotoxicity of the solvent vehicle for cyclosporine. *J Pharmacol Exp Ther* **268**: 1051–1056

Younes A, Sarris A, Melnyk A, Romaguera J, McLaughlin P, Swan F, Rodriguez MA, Hagemeister F, Moore D, North L, Smith TL and Cabanillas F (1995) Three-hour paclitaxel infusion in patients with refractory and relapsed non-Hodgkin's lymphoma. *J Clin Oncol* **13**: 583–587

*© 2001 Cancer Research Campaign*
